# P32 Bloodstream infection following transrectal ultrasound-guided prostate biopsy: antibiotic susceptibility of isolates relative to perioperative antibiotic prophylaxis regimen

**DOI:** 10.1093/jacamr/dlae136.036

**Published:** 2024-08-23

**Authors:** C Mulrooney, S Gregg, M Leonard, M O’Loughlin, C Dowling, U Ni Riain

**Affiliations:** Microbiology Department, University Hospital Galway, Galway, Ireland; Microbiology Department, University Hospital Galway, Galway, Ireland; Microbiology Department, University Hospital Galway, Galway, Ireland; Urology Department, University Hospital Galway, Galway, Ireland; Urology Department, University Hospital Galway, Galway, Ireland; Microbiology Department, University Hospital Galway, Galway, Ireland

## Abstract

**Background:**

Transrectal ultrasound (TRUS)-guided biopsy of the prostate is a standard investigation for the diagnosis of prostate cancer.^1^ Perioperative antibiotic prophylaxis is standard of care, notwithstanding which, infection complications post biopsy are reported at 2%–6%.^2,3^ Single agent ciprofloxacin was used as perioperative antibiotic prophylaxis for TRUS-guided biopsy within our centre until 2011 when data relating increasing rates of fluoroquinolone resistance among Enterobacterales prompted a change. From 2011, prophylaxis changed to dual agent ciprofloxacin plus gentamicin. Scheduled review of the hospital’s empirical antimicrobial guidelines is undertaken every 3 years, most recently in 2024. Review of antibiograms of bloodstream infection isolates is an integral part of the antimicrobial guideline review process, to inform change in empirical regimens, if indicated, based on local antibiotic resistance rates.

**Objectives:**

Specific to TRUS-guided biopsy guidelines, antibiotic susceptibility data for organisms associated with invasive infection post TRUS biopsy from 2021 to 2023 was reviewed.

**Methods:**

Post-procedure surveillance database identified patients who required hospital readmission due to suspected or confirmed infection following TRUS-guided biopsy of prostate between January 2021 and December 2023. Laboratory information systems of all five hospitals in the region were accessed for blood culture results and the organism and antibiogram of any associated positive blood culture was recorded.

**Results:**

Of 2073 patients who underwent TRUS-guided biopsy, 63 (3.04%) patients were readmitted to hospital with suspected infection. Of these readmitted patients, 13/63 had positive blood cultures. All of the isolates (13/13, 100%) were *Escherichia coli* and 12 of 13 (92.3%) *E. coli* isolates tested susceptible or susceptible increased exposure to at least one of gentamicin or ciprofloxacin: 12/13 (92.3%) susceptible to gentamicin; 8 (61.5%) susceptible/susceptible increased exposure to ciprofloxacin; 1 (7.7%) resistant to both ciprofloxacin and gentamicin.

**Conclusions:**

The centre’s rate of significant infection complication is in line with published rates. The revised perioperative antibiotic prophylaxis regimen of dual therapy with gentamicin and ciprofloxacin remains appropriate. Following 2073 TRUS-guided biopsies over a 3 year period, only one patient requiring readmission with infection had a bloodstream infection with an organism resistant to both prophylactic agents, while five patients had bloodstream infection with an organism resistant to ciprofloxacin, the previous monotherapy regimen.
